# A candidate-set-free algorithm for generating *D*-optimal split-plot designs

**DOI:** 10.1111/j.1467-9876.2007.00581.x

**Published:** 2007-05

**Authors:** Bradley Jones, Peter Goos

**Affiliations:** SAS InstituteCary, USA; Universiteit AntwerpenBelgium

**Keywords:** *D*-optimality, Exchange algorithm, Hard-to-change factors, Multistratum design, Split-plot design, Tailor-made design

## Abstract

We introduce a new method for generating optimal split-plot designs. These designs are optimal in the sense that they are efficient for estimating the fixed effects of the statistical model that is appropriate given the split-plot design structure. One advantage of the method is that it does not require the prior specification of a candidate set. This makes the production of split-plot designs computationally feasible in situations where the candidate set is too large to be tractable. The method allows for flexible choice of the sample size and supports inclusion of both continuous and categorical factors. The model can be any linear regression model and may include arbitrary polynomial terms in the continuous factors and interaction terms of any order. We demonstrate the usefulness of this flexibility with a 100-run polypropylene experiment involving 11 factors where we found a design that is substantially more efficient than designs that are produced by using other approaches.

## 1. Introduction

Split-plot designs arise in experimental studies when a completely randomized run order is structurally impossible, expensive or inconvenient. So, a statistician's recommendation to randomize the order of the runs is often ignored in practice. Instead the experimenter rearranges the runs of the design so that hard-to-change factors only need to be changed a few times over the course of the study. This rearrangement, though seemingly innocuous, creates a split-plot structure, i.e. the experiment is performed in groups of runs where the hard-to-change factors stay constant within each group.

The terminology for split-plot designs comes from their original application in agricultural experiments where a factor that only varies between separate plots of land is called a whole-plot factor. This is because the researcher applies only one of its possible levels to the *whole plot*. A factor whose levels vary within each plot is called a subplot factor. In industrial application the hard-to-change factors are whole-plot factors and the groups of runs are the whole plots. A subplot factor is one that is easy to reset from run to run.

Though the primary motivation for doing split-plot experiments is economic or logistic necessity, there are also statistical reasons to prefer a split-plot arrangement over a completely randomized design in some cases. Varying a hard-to-change factor can cause a substantial disruption in the process. This results in a random change in the mean of the process response from one group of runs to the next. If the process is completely reset between each run, this random effect of varying the hard-to-change factors gets added to the usual process variance. This makes detection of the effect of a change in any factor more difficult. By contrast, in a split-plot design, only the whole-plot effects experience this extra variability. Support for this argument in favour of using split-plot experiments has been provided by Anbari and Lucas (1994), who showed that some arrangements of two-level factorial designs in whole plots lead to smaller prediction variances than the corresponding completely randomized design, and Goos and Vandebroek (2001, 2004), who also demonstrated that completely randomized designs can be outperformed in terms of *D*-efficiency.

The design of industrial split-plot experiments received attention in Addelman (1964) and Anbari and Lucas (1994), who discussed the arrangement of factorial designs in a split-plot format, Letsinger *et al.* (1996), who investigated the efficiency of various second-order designs when run as a split-plot experiment, and Draper and John (1998), who discussed modifications of central composite designs and Box–Behnken designs to be run in a split-plot format. The design of two-level fractional factorial split-plot experiments has been discussed in Huang *et al.* (1998),Bingham and Sitter (1999, 2001) and Bingham *et al.* (2004) whereas 24-run two-level split-plot designs have been presented in Kowalski (2002). Kulahci and Bisgaard (2005) showed how Plackett–Burman designs can be used to construct split-plot designs. A sequential strategy for designing multistratum response surface designs, special cases of which are split-plot designs, was presented by Trinca and Gilmour (2001). The optimal design of first- and second-order split-plot experiments later received attention by Goos and Vandebroek (2001, 2003, 2004). The optimal design of split-plot experiments for spherical design regions received special attention in Mee (2007). Standard experimental designs for split-plot mixture process variable designs were proposed by Kowalski *et al.* (2002). Optimal designs for this type of experiment are reported in Goos and Donev (2007).

A recent line of references focuses on the arrangement of standard response surface designs like central composite and Box–Behnken designs in a split-plot format so that ordinary least squares estimation provides the same estimates as generalized least squares estimation (see Vining *et al.* (2005) and Parker *et al.* (2007a,b));. A key feature of some of these ‘equivalent estimation’ split-plot designs is that they allow a model-independent estimation of the variance components that are needed for statistical inference. An overview of some of the recent work on the design of split-plot experiments, including a comparison of optimal to equivalent estimation designs, has been given in Goos (2002, 2006).

The previously referenced literature on the design of split-plot experiments mainly addresses the question of how to superimpose a split-plot structure safely on well-known designs. Another line of research focuses on the algorithmic construction of split-plot designs to allow more flexibility in the choice of sample size, whole-plot size and *a priori* model. Trinca and Gilmour (2001) presented a sequential algorithmic approach to construct multistratum experiments in general and split-plot experiments in particular. Their algorithm allows researchers to combine the points of given designs for the factors that are applied to the different strata, for instance the hard-to-change factors and the easy-to-change factors, in an efficient way. When using the Trinca and Gilmour (2001) algorithm, the designs in the hard-to-change factors and the easy-to-change factors can be optimal designs or modified central composite designs, for example. The algorithms of Goos and Vandebroek (2001, 2003, 2004) for constructing tailor-made split-plot designs are not sequential and use the *D*-optimality criterion to select simultaneously the combinations of the hard-to-change and the easy-to-change factors and to arrange the selected combinations, or runs, in whole plots.

A difficulty with the algorithms of Goos and Vandebroek (2001, 2003, 2004) is that they require the construction of a candidate set, which is the set of allowable combinations of factor levels. The algorithms then select the factor level combinations and arrange them in whole plots so that the *D*-optimality criterion is maximized. This approach, which became traditional in the literature on the algorithmic construction of optimal designs after the publication of the first point exchange algorithm by Fedorov (1972), can be problematic when the number of experimental factors is large and/or the experimental region is highly constrained. This is because a candidate set that covers the entire design region well in such cases requires a large number of factor level combinations. For example, a good candidate set for a split-plot polypropylene experiment involving seven hard-to-change factors and four easy-to-change factors, one of which is categorical, has at least 10 368 points, which complicates the search for an optimal design by using a point exchange algorithm.

The purpose of this paper is to present an algorithmic approach to constructing tailor-made split-plot experiments without having to specify a candidate set. After the introduction of the statistical model, the candidate-set-free algorithm is outlined in Section 3 and compared with previous algorithms in Section 4. The good performance of the candidate-set-free algorithm is first demonstrated by using a proof-of-concept example. Next, we apply the new algorithm to a polypropylene experiment and compare the resulting design with the experiment that was actually conducted and a sequential approach that uses the algorithm of Trinca and Gilmour (2001).

## 2. Statistical model and analysis

For a split-plot experiment with sample size *n* and *b* whole plots, the model can be written as 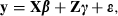
(1) where **X** represents the *n*×*p* model matrix containing the settings of both the whole-plot factors **w** and the subplot factors **s** and their model expansions, ***β*** is a *p*-dimensional vector containing the *p* fixed effects in the model, **Z** is an *n*×*b* matrix of 0s and 1s assigning the *n* runs to the *b* whole plots, ***γ*** is the *b*-dimensional vector containing the random effects of the *b* whole plots and ***ɛ*** is the *n*-dimensional vector containing the random errors. It is assumed that 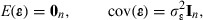
(2)
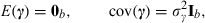
(3)
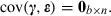
(4)

Under these assumptions, the covariance matrix of the reponses, var(**y**), is 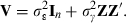
(5)

When the entries of **y** are arranged per whole plot, then 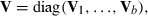
(6) where 
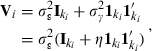
(7)*k*_*i*_ is the number of observations in the *i*th whole plot, and the variance ratio 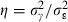 is a measure for the extent to which observations within the same whole plot are correlated. The larger *η*, the more the observations within one whole plot are correlated.

When the random-error terms as well as the whole-plot effects are normally distributed, the maximum likelihood estimator of the unknown model parameter ***β*** in model (1) is the generalized least squares estimator 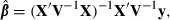
(8) with covariance matrix 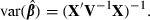
(9)

The information matrix on the unknown fixed parameters ***β*** is given by 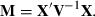
(10)

A commonly used criterion to select experimental designs is the *D*-optimality criterion which seeks designs that maximize the determinant of the information matrix. We use *D*-efficiency to compare the quality of two designs with information matrices **M**_1_ and **M**_2_. *D*-efficiency is defined as 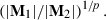


In general, the optimal split-plot design will depend on the variance ratio *η* through **V**. The *D*-optimality criterion has been used for constructing split-plot designs by Goos and Vandebroek (2001, 2003, 2004) and it is also the criterion which is implemented in the candidate-set-free algorithm that is described in the next section.

## 3. Algorithm

The algorithm below is novel because, unlike point exchange algorithms, it avoids the need for the explicit construction of a candidate set. This section provides a rough general description of our candidate-set-free algorithm for generating *D*-optimal split-plot designs. Detailed pseudocode is provided in Appendix A.

The algorithm requires the prior specification of the following:

- for each factor whether it is continuous, categorical or a mixture ingredient,
- designation of the factors that are hard to change,
- any additional constraints on factor combinations,
- the number *b* and size of the whole plots (which yields the sample size),
- the ratio *η* of the whole-plot to the error variance,
- the *a priori* model and
- the number of starting designs or tries *t* to consider.

Given this information, the body of the algorithm has two parts. The first part is the creation of a starting design. The second is the iterative improvement of this design until no further improvement is possible. Improvements are measured by increases in the objective function 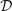
=|**M**|=|**X**^′^**V**^−1^**X**|. The two parts are performed *t* times. Each time the value of 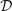 that is found in the current iterate is compared with the maximum value of 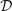 from all the previous iterates. If the current value is higher, then it becomes the new maximum and the current design is stored.

The starting design is formed column by column. For subplot factor columns, the values for each row are chosen randomly. For whole-plot factor columns, *b* random numbers are chosen. All the rows in a given whole plot have the same value. This procedure gives the starting design the desired split-plot structure.

Improvements are made to the starting design by considering changes in the design element by element. This is inspired by Meyer and Nachtsheim (1995). The procedure for changing any given element depends on whether that element is in a subplot factor column or a whole-plot factor column.

For an element in a subplot factor column, the objective function is evaluated over a discrete number of values spanning the range of that factor. If the maximal value of the objective function is larger than the current maximum, then the current maximum is replaced and the current element in the design is replaced by the factor setting corresponding to the maximal value.

For an element in a whole-plot factor column the procedure is more involved. If this element changes then all the elements in the same whole plot for that column must also change. A discrete number of values for that whole-plot value are considered. Again, if the maximal value of the objective function is larger than the current maximum, then the current maximum is replaced and all the rows in that whole plot are replaced by the factor setting corresponding to the maximal value.

This element-by-element procedure continues until a complete cycle through the entire design has been completed. Then, another complete cycle through the design is performed noting whether any element changes in the current pass. This continues until no changes are made in a whole pass or until a specified maximum number of passes have been executed.

## 4. Evaluation of the candidate-set-free algorithm

The purpose of this section is to compare the candidate-set-free algorithm with alternative methods for constructing *D*-optimal designs. A comparison is made in terms of ease of use, quality of the design produced and computing time.

### 4.1. Ease of use

The main advantage of the candidate-set-free algorithm that was presented in Section 3 is that it does not require the user to specify a candidate set. For simple design problems, such as problems involving unconstrained continuous factors only and a cuboidal experimental region, good candidate sets for first- and second-order models are given by the points of two- or three-level factorial designs respectively. For other design problems, involving constrained continuous and/or mixture variables, constructing a good candidate set may be difficult and time consuming. The construction of good candidate sets for such problems requires experience and is, for spherical design regions, even a matter of on-going research (see Mee (2007)). Since running design construction algorithms using poor candidate sets leads to inefficient designs, the fact that the candidate-set-free algorithm does not require a candidate set is an important practical advantage over the algorithm of Goos and Vandebroek (2003) and sequential algorithmic approaches like that in Trinca and Gilmour (2001).

The fact that the construction of a good candidate set often takes more time than running a classical algorithm for computing an optimal design is not captured by the timing study that is reported in Section 4.3. Obviating the need for the construction of a candidate set, however, is a major contribution of the candidate-set-free algorithm.

### 4.2. Design efficiency

We have computed *D*-optimal designs for various design problems, including the examples in Goos and Vandebroek (2003), using the candidate-set-free algorithm, the algorithm of Goos and Vandebroek (2003) and two sequential approaches. In the sequential approaches, one of which was proposed by Trinca and Gilmour (2001) and which are illustrated in detail in Sections 6.3 and 6.4.1, the designs for the whole-plot factors and the subplot factors are constructed sequentially. Compared with the algorithm of Goos and Vandebroek (2003), the sequential approaches offer the advantage that two smaller candidate sets can be used instead of one large candidate set when the design problem involves a large number of factors. This is attractive from a computational point of view. Unfortunately, the sequential approaches to constructing *D*-optimal split-plot designs do not always lead to highly efficient designs, even when the designs for the whole-plot factors and the subplot factors are both constructed by using the *D*-optimality criterion. This is illustrated in Section 6.4.2, where the sequential approaches yield designs that are more than 10% less efficient than the design that is produced by the candidate-set-free algorithm. When the goal is to construct *D*-optimal split-plot designs, we would therefore not recommend a sequential approach in general.

The candidate-set-free algorithm and the algorithm of Goos and Vandebroek (2003) are close competitors in terms of efficiency of the designs generated provided that the latter algorithm is run with a good candidate set. For first-order models in the absence of constraints on the factor levels, the two algorithms produce equivalent designs. For second-order models and for design problems involving constraints on the factor levels, applying the candidate-set-free algorithm for a given computing time leads to designs that perform up to 0.5% better in terms of *D*-optimality than the algorithm of Goos and Vandebroek (2003). These small gains in efficiency are due to the nearly continuous optimization that can be performed with the candidate-set-free algorithm. Such a nearly continuous optimization could also be done by letting the algorithm of Goos and Vandebroek (2003), which performs a discrete optimization, be followed by an adjustment algorithm which is similar to that suggested by Donev and Atkinson (1988).

### 4.3. Computing time

The fact that the algorithm of Goos and Vandebroek (2003) uses a candidate set and exchange and interchange steps makes it computationally more intensive than the candidate-set-free algorithm. The candidate set grows exponentially with the number of factors in an experiment. Considering all exchanges between rows of the candidate set and rows of the design makes such an algorithm run in exponential time in the number of factors. By contrast, our candidate-set-free algorithm runs in polynomial time in the number of factors and the number of levels considered for each factor. For a fixed number of factors, the candidate-set-free algorithm can allow for a much finer discretization of the range of each factor for the same computational cost as an algorithm using a candidate set, leading to the small efficiency improvements that were mentioned in Section 4.2.

## 5. Theoretical example

Consider a problem with two whole-plot factors and five subplot factors. Suppose that there are resources for 24 runs to be performed in eight whole plots of three runs each. All the factors have two levels.

If the number of runs and the number of runs per whole plot are both powers of 2, then Bingham and Sitter (1999, 2001) and Bingham *et al.* (2004) have shown how to choose generators for the whole-plot and subplot factors to maintain orthogonality while imposing the desired split-plot structure.

The difficulty with the example under consideration is that the number of runs per whole plot is 3 (not a power of 2). As a result, within each whole plot it is impossible to balance the settings of the subplot factors while maintaining a two-level design. A 24-run Plackett–Burman design with seven columns might serve as a template for solving this problem. One could sort such a design by any two columns to create the desired split-plot structure. However, the resulting covariance matrix of the coefficients will not be diagonal in general. So, the estimates of the coefficients will be correlated unnecessarily.

Table 1 shows a globally optimal design, constructed by using our new algorithm, for estimating a main effects model. Note that the values of the two whole-plot factors over the eight whole plots are a replicated 2^2^ factorial design. Within each whole plot the sum of the values of each subplot factor is always either −1 or 1; moreover, the inner product of any pair of columns is 0. So, the design is as balanced as it possibly can be given that each whole plot is only three runs instead of four.

**Table 1:** Optimal 24-run split-plot design in eight whole plots of size 3

*Whole plot*	*w*_1_	*w*_2_	*s*_1_	*s*_2_	*s*_3_	*s*_4_	*s*_5_
1	1	1	1	−1	1	1	1
1	1	1	−1	−1	−1	−1	−1
1	1	1	−1	1	1	−1	1
2	−1	1	1	−1	1	−1	−1
2	−1	1	−1	1	1	−1	1
2	−1	1	1	1	−1	1	−1
3	1	−1	1	−1	−1	1	1
3	1	−1	1	1	−1	−1	−1
3	1	−1	−1	−1	1	−1	−1
4	−1	−1	−1	−1	1	1	−1
4	−1	−1	−1	−1	−1	1	−1
4	−1	−1	1	1	1	−1	1
5	1	1	−1	1	−1	−1	−1
5	1	1	−1	1	−1	1	1
5	1	1	1	−1	1	1	−1
6	−1	1	1	1	1	1	−1
6	−1	1	1	−1	−1	−1	1
6	−1	1	−1	−1	−1	1	1
7	1	−1	1	1	1	1	1
7	1	−1	1	−1	−1	−1	1
7	1	−1	−1	1	1	1	−1
8	−1	−1	−1	−1	1	−1	1
8	−1	−1	1	1	−1	−1	−1
8	−1	−1	−1	1	−1	1	1

The information matrix of the design, assuming that the ratio of the whole-plot variance to the error variance (*η*) and 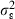 are both 1, is in Table 2. The matrix is diagonal so estimates of the model coefficients are uncorrelated. Note that the diagonal elements that are associated with the intercept and whole-plot factor effects are 6. By contrast, the elements that are associated with the subplot effects are 22. We could view these values as effective sample sizes for estimating these effects. The individual variances of the coefficients are proportional to the reciprocals of the diagonal elements of the matrix. The variances of the whole-plot factor effects (and intercept) are thus much larger than the variances of the subplot factor effects. This is a direct result of the split-plot structure. Again, assuming that *η* and 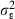 are 1 and that a completely randomized design were run, then the values on the diagonal of the information matrix would be 12. Thus, for this variance ratio, the split-plot design estimates the main effects of the two whole-plot factors with twice the variance of a completely randomized design but those of the five subplot factors with only 6/11ths the variance. Thus, overall, the split-plot design is more efficient.

**Table 2:** Diagonal information matrix for the split-plot design in Table 1

*I*	*w*_1_	*w*_2_	*s*_1_	*s*_2_	*s*_3_	*s*_4_	*s*_5_
6	0	0	0	0	0	0	0
0	6	0	0	0	0	0	0
0	0	6	0	0	0	0	0
0	0	0	22	0	0	0	0
0	0	0	0	22	0	0	0
0	0	0	0	0	22	0	0
0	0	0	0	0	0	22	0
0	0	0	0	0	0	0	22

## 6. Polypropylene experiment

### 6.1. Background

In 2004 and 2005, four Belgian companies, Domo PolyPropylene Compounds (a producer of thermoplastic materials), Europlasma (a developer of gas plasma systems), Structuplas (a company that specialized in the finishing of thermoplastic materials) and Techni-Coat International (a company that specialized in applying coatings) ran a large experiment to investigate the effect of several additives and a gas plasma surface treatment on the adhesive properties of polypropylene. The experiment was of great interest to car manufacturers who are increasingly using polypropylene because it is inexpensive and light, and because it can be recycled. The experiment was therefore financially supported by Flanders’ Drive, a technological platform that stimulates innovation in the automotive industry in Flanders (the northern part of Belgium) and that itself is supported by the Flemish Government.

An undesirable property of polypropylene is that glues and coatings do not adhere well to its surface unless it undergoes a surface treatment, like a gas plasma treatment. The goal of the experiment was to search for economical plasma treatments that lead to good adhesion. For that, several experimental factors related to the plasma treatment: the gas flow rate, the power, the reaction time and the type of gas that was used. Three types of gas were utilized in the experiment: one etching gas and two activation gases. After some pilot tests, the engineer who was in charge of the plasma treatment decided to study gas flow rates between 1000 and 2000 sccm, powers ranging from 500 to 2000 W and reaction times between 2 and 15 min.

### 6.2. The design problem

On the basis of a literature study and the engineers’ experience with applying coatings and glues to various kinds of plastics and with gas plasma treatments, a slack variable statistical model was selected. This choice seemed justified because polypropylene is inactive with respect to adhesion. Also, three of the seven additives have very small ranges so a Scheffé mixture model would force the analyst to deal with severe collinearity. Cornell (2002), section 6.13, compared the two approaches and cautioned against the use of slack variable models. Besides an intercept, the model that was used here included

- the main effects of the seven additives,
- the six two-factor interactions involving EPDM and each of the other additives,
- the main effects of the gas type, the flow rate, the power and the reaction time,
- all two-factor interactions of these four factors,
- the quadratic effects of the flow rate, the power and the reaction time, and
- all two-factor interactions between the seven additives, on the one hand, and the four plasma treatment factors, on the other hand.

As a result, the number of fixed parameters to be estimated, *p*, equalled 66.

For estimating the model, the grant for Europlasma, Structuplas and Techni-Coat International allowed for 100 runs. However, it was impossible for Domo PolyPropylene Compounds to prepare 100 different polypropylene formulations as this was very labour intensive and the minimum batch size was quite large. A reasonable compromise was to have Domo *PolyPropylene* Compounds produce 20 different batches or formulations of polypropylene and to use the material in those batches to test the effects of 100 different gas plasma treatments on the adhesion of glues and coatings.

Since each formulation or batch was used for multiple experimental runs, the result is a split-plot design. The seven additives are fixed for each batch, so they are ‘hard-to-change’ or whole-plot factors. They are labelled *w*_1_,…,*w*_7_ in Table 3. The levels of the factors that are related to the gas plasma treatment were reset independently for each run, so these factors are subplot factors. They are labelled *s*_1_,…,*s*_4_ in Table 3.

**Table 3:** Ranges and levels of factors that were studied in the polypropylene experiment

*Factor*	*Range or level*
EPDM (*w*_1_)	0–15%
Ethylene (*w*_2_)	0–10%
Talc (*w*_3_)	0–20%
Mica (*w*_4_)	0–20%
Lubricant (*w*_5_)	0–1.5%
UV stabilizer (*w*_6_)	0–0.8%
Ethylene vinyl acetate (*w*_7_)	0–1.5%
Flow rate (*s*_1_)	1000–2000 sccm
Power (*s*_2_)	500–2000 W
Reaction time (*s*_3_)	2–15 min
Gas type (*s*_4_)	Etching gas
	Activation gas 1
	Activation gas 2

All the design approaches that are described below use the *D*-optimality criterion as there were many practical considerations, each of which rendered the use of standard design methods impossible:

- the presence of the multicomponent constraint,
- a categorical subplot factor at three levels,
- a whole-plot size of 20,
- the interest in all the two-factor interactions involving EPDM,
- the overall sample size of 100 and
- the need to estimate quadratic effects.

### 6.3. The original experimental design

The experimental design that was actually conducted by the four companies was constructed in a sequential fashion to satisfy the constraint that only 20 different batches of polypropylene could be used. This was done using the SAS procedure.

First, 20 polypropylene formulations were selected by computing a *D*-optimal completely randomized design with 20 runs for a model involving the main effects of the seven additives and all two-factor interactions involving EPDM, taking into account the multicomponent constraint for the additives talc and mica. The resulting design is given in Table 4.

**Table 4:** Polypropylene formulations utilized in the original polypropylene experiment (in coded form) along with the number of runs (*k*_*i*_) using each of them

*Whole plot*	*w*_1_	*w*_2_	*w*_3_	*w*_4_	*w*_5_	*w*_6_	*w*_7_	*k*_*i*_
1	−1	−1	−1	−1	−1	−1	1	7
2	−1	−1	−1	1	1	−1	−1	6
3	−1	−1	1	−1	−1	1	−1	6
4	−1	−1	1	−1	1	1	1	5
5	−1	1	−1	−1	1	1	−1	7
6	−1	1	−1	1	−1	1	1	7
7	−1	1	1	−1	−1	−1	−1	4
8	−1	1	1	−1	1	−1	1	5
9	1	−1	−1	−1	−1	−1	1	3
10	1	−1	−1	−1	1	1	1	6
11	1	−1	−1	1	−1	−1	−1	6
12	1	−1	−1	1	1	1	1	5
13	1	−1	1	−1	−1	1	−1	3
14	1	−1	1	−1	1	−1	−1	4
15	1	1	−1	−1	−1	1	−1	4
16	1	1	−1	−1	1	−1	−1	6
17	1	1	−1	1	−1	−1	1	4
18	1	1	−1	1	1	1	−1	4
19	1	1	1	−1	−1	1	1	5
20	1	1	1	−1	1	−1	1	3

The 20 polypropylene formulations were combined with an 81-point candidate set for the gas plasma factors to construct a 1620-element candidate set for computing a *D*-optimal design for estimating the full 66-parameter model in the additives and the gas plasma factors. The resulting design is displayed in Table 5 and is available in electronic form from the authors. The design that was obtained in this fashion exhibits a lack of balance because not every batch was used equally often in the design. The numbers of runs for each batch, *k*_*i*_, are displayed in the last column of Table 4. It can be verified that the minimum whole-plot size equals 3, whereas the maximum whole-plot size is 7. The number of batches being used three, four, five, six and seven times was 3, 5, 4, 5 and 3 respectively. The heterogeneity of the whole-plot sizes was inconvenient as it required Domo PolyPropylene Compounds to produce batches of different sizes. Nevertheless, this was how the original experiment was run.

**Table 5:** Original design for the polypropylene experiment

*Whole plot*	*s*_1_	*s*_2_	*s*_3_	*s*_4_	*Whole plot*	*s*_1_	*s*_2_	*s*_3_	*s*_4_
1	−1	1	1	C	10	0	0	0	C
1	1	−1	−1	C	10	1	1	−1	C
1	−1	1	−1	B	10	−1	−1	1	B
1	1	−1	0	B	10	1	−1	−1	B
1	−1	−1	0	A	10	−1	0	−1	A
1	1	1	−1	A	10	1	0	1	A
1	1	1	1	A	11	1	1	0	C
2	−1	1	−1	C	11	−1	−1	0	B
2	0	−1	1	C	11	1	0	−1	B
2	0	1	1	B	11	−1	1	−1	A
2	1	−1	0	B	11	1	−1	−1	A
2	−1	−1	1	A	11	1	0	1	A
2	1	1	0	A	12	−1	1	1	C
3	−1	0	1	C	12	0	−1	−1	C
3	1	−1	−1	C	12	−1	0	0	B
3	0	−1	1	B	12	1	1	1	B
3	0	1	−1	B	12	1	1	−1	A
3	−1	−1	−1	A	13	0	1	1	C
3	1	1	1	A	13	1	−1	0	B
4	1	0	0	C	13	0	0	0	A
4	−1	−1	−1	B	14	−1	−1	0	C
4	1	1	0	B	14	−1	1	1	B
4	−1	1	1	A	14	1	0	−1	B
4	0	−1	0	A	14	0	0	−1	A
5	−1	−1	1	C	15	−1	−1	−1	B
5	1	1	1	C	15	−1	1	1	B
5	0	0	−1	B	15	0	−1	1	A
5	1	0	1	B	15	1	1	−1	A
5	−1	1	−1	A	16	−1	0	−1	C
5	0	0	1	A	16	1	−1	1	C
5	1	−1	−1	A	16	−1	0	1	B
6	−1	−1	−1	C	16	0	1	0	B
6	0	1	0	C	16	−1	−1	−1	A
6	1	−1	1	C	16	−1	1	1	A
6	−1	0	1	B	17	−1	0	1	C
6	1	0	−1	B	17	−1	1	−1	B
6	0	−1	−1	A	17	0	−1	0	B
6	0	1	1	A	17	0	0	0	A
7	0	−1	0	C	18	0	0	−1	C
7	−1	1	0	B	18	−1	1	−1	B
7	1	1	1	B	18	1	−1	1	B
7	1	0	0	A	18	−1	−1	0	A
8	0	1	1	C	19	−1	1	−1	C
8	−1	−1	−1	B	19	1	0	1	C
8	1	1	−1	B	19	0	0	0	B
8	−1	1	−1	A	19	−1	−1	1	A
8	1	−1	1	A	19	1	−1	−1	A
9	1	0	1	B	20	1	−1	−1	C
9	−1	1	1	A	20	1	−1	1	B
9	1	−1	0	A	20	1	1	1	A

### 6.4. Alternative design strategies

The approach that was used in the original experiment might be improved in several ways. First, the design construction method did not allow the researchers to force the whole-plot sizes to be equal. Second, the selection and the arrangement of the design points were not done by taking into account the split-plot correlation structure of the experiment. One way to circumvent these problems is to use the sequential approach that was presented by Trinca and Gilmour (2001) to construct a split-plot design with 20 whole plots of size 5. Alternatively, the candidate-set-free algorithm could be utilized to construct a 100-run *D*-optimal split-plot design with whole-plot sizes equal to 5.

#### 6.4.1. The algorithm of Trinca and Gilmour (2001)

The approach that was advocated by Trinca and Gilmour (2001) is sequential in nature because it involves selecting the combinations of levels for the whole-plot and subplot factors in the experiment first and arranging them in a split-plot design with the desired whole-plot structure next. The algorithm arranges the subplot factor level combinations to be nearly orthogonal to the whole plots.

The sequential approach has several advantages. First, the resulting designs do not depend on prior specification of the variance components of the split-plot model. Second, the construction method does not require the specification of a large candidate set of whole-plot and subplot factor level combinations. This makes the approach attractive for designing the polypropylene experiment which involves seven whole-plot factors and four subplot factors.

As explained in Section 6.3, the *D*-optimality criterion selected the 20 formulations that are displayed in Table 4. This design is the first building-block that we used as an input to the algorithm of Trinca and Gilmour (2001).

The second building-block that is needed as an input to the algorithm is a 100-run design for the four subplot factors. We selected a *D*-optimal 100-point design for the three continuous subplot factors and the categorical factor. The design that was produced by the algorithm of Trinca and Gilmour is displayed in Table 6. Assuming an *η*-value of 1, the relative efficiency of this design relative to the original design in Table 5 is 116.5%, indicating a substantial improvement.

**Table 6:** Design obtained by using the sequential approach of Trinca and Gilmour (2001)

*Whole plot*	*s*_1_	*s*_2_	*s*_3_	*s*_4_	*Whole plot*	*s*_1_	*s*_2_	*s*_3_	*s*_4_
1	1	−1	1	A	11	−1	−1	−1	B
1	1	−1	−1	B	11	−1	0	1	C
1	1	0	0	C	11	0	1	0	A
1	−1	−1	1	B	11	1	−1	−1	C
1	1	1	1	B	11	1	−1	1	A
2	−1	−1	1	A	12	1	1	1	C
2	−1	1	−1	A	12	0	0	1	A
2	−1	1	1	C	12	−1	1	1	B
2	0	−1	−1	C	12	−1	−1	0	C
2	1	0	0	B	12	1	−1	1	B
3	−1	−1	1	C	13	−1	0	−1	A
3	1	0	1	B	13	0	1	0	C
3	0	−1	−1	A	13	1	1	−1	B
3	1	1	−1	C	13	−1	1	1	B
3	−1	1	0	B	13	1	1	1	A
4	−1	1	−1	B	14	0	1	1	A
4	1	−1	−1	C	14	−1	0	1	B
4	1	1	−1	A	14	−1	1	−1	C
4	−1	1	1	C	14	1	−1	1	C
4	−1	−1	0	A	14	1	1	−1	B
5	−1	−1	1	A	15	−1	1	1	C
5	0	0	1	C	15	0	−1	1	B
5	1	−1	0	B	15	−1	−1	−1	B
5	1	1	1	A	15	1	−1	−1	C
5	−1	1	1	B	15	1	1	−1	B
6	−1	1	1	A	16	1	−1	−1	A
6	−1	−1	1	B	16	−1	−1	0	C
6	0	−1	1	C	16	−1	1	−1	A
6	−1	1	−1	C	16	1	1	−1	C
6	−1	−1	−1	A	16	−1	0	−1	B
7	1	−1	0	A	17	0	1	1	B
7	0	1	−1	B	17	1	1	1	C
7	−1	−1	−1	C	17	−1	1	0	A
7	−1	1	1	A	17	−1	0	−1	C
7	1	1	1	C	17	1	1	−1	A
8	−1	−1	−1	B	18	1	0	0	A
8	1	−1	1	B	18	0	1	−1	C
8	−1	0	1	A	18	1	−1	−1	B
8	−1	1	0	B	18	1	1	1	B
8	1	1	−1	A	18	1	−1	1	C
9	1	1	1	A	19	−1	1	−1	B
9	1	−1	1	C	19	−1	−1	1	B
9	−1	1	−1	C	19	1	1	1	B
9	1	0	−1	A	19	1	−1	−1	B
9	−1	−1	1	A	19	0	−1	−1	A
10	1	−1	−1	A	20	1	−1	1	A
10	0	0	−1	B	20	0	−1	0	B
10	−1	−1	−1	C	20	−1	−1	−1	A
10	1	1	0	C	20	−1	−1	1	C
10	−1	1	−1	A	20	1	0	−1	C

#### 6.4.2. The candidate-set-free algorithm

Another alternative is to use the candidate-set-free algorithm that was outlined in Section 3. Sequential approaches restrict the solution space, thus making it impossible to find locally optimal designs in general. But, unlike the sequential approaches that were described above, the candidate-set-free algorithm solves the optimization problem for the whole-plot and subplot factors simultaneously. This makes it more likely to find a locally optimal design.

The *D*-optimal design that was produced by the candidate-set-free algorithm in the JMP6 software for *η*=1 is displayed in Table 7. Its 20 whole-plot factor levels are given in Table 8. Producing that design required three tries taking between 10 and 20 min each on a 1.6 GHz processor.

**Table 8:** Polypropylene formulations utilized in the *D*-optimal design in Table 7

*Whole plot*	*w*_1_	*w*_2_	*w*_3_	*w*_4_	*w*_5_	*w*_6_	*w*_7_
1	1	−1	−1	−1	1	1	−1
2	1	−1	1	−1	1	−1	1
3	−1	1	−1	−1	1	1	−1
4	−1	−1	−1	1	1	1	1
5	−1	1	1	−1	−1	−1	1
6	−1	−1	−1	1	−1	1	−1
7	−1	−1	1	−1	1	−1	−1
8	1	1	−1	1	1	−1	−1
9	1	1	1	−1	1	1	1
10	−1	1	−1	1	1	−1	−1
11	1	−1	−1	−1	1	−1	1
12	−1	−1	1	−1	−1	1	−1
13	1	−1	1	−1	−1	−1	−1
14	1	1	−1	−1	−1	1	1
15	−1	1	−1	1	−1	−1	1
16	1	1	1	−1	−1	1	−1
17	1	−1	−1	1	−1	1	1
18	−1	−1	−1	−1	−1	−1	1
19	1	1	−1	−1	−1	−1	−1
20	−1	1	1	−1	1	1	1

**Table 7:** *D*-optimal design obtained by using the candidate-set-free algorithm outlined in Section 3

*Whole plot*	*s*_1_	*s*_2_	*s*_3_	*s*_4_	*Whole plot*	*s*_1_	*s*_2_	*s*_3_	*s*_4_
1	−1	1	−1	C	11	1	1	1	B
1	1	−1	1	C	11	1	1	−1	A
1	−1	−1	−1	A	11	−1	−1	−0.1	B
1	0.1	1	1	A	11	−1	1	1	C
1	1	−1	−1	B	11	1	−1	−1	C
2	1	1	0.2	C	12	1	1	1	B
2	−1	1	1	B	12	1	1	−1	A
2	1	−1	−1	B	12	1	−1	1	C
2	−1	−1	1	A	12	−0.1	−1	−1	B
2	−1	1	−1	A	12	−1	−0.3	1	A
3	−1	−1	1	A	13	1	−0.2	1	B
3	−1	−1	−1	C	13	−1	1	1	C
3	−1	1	1	B	13	−1	1	−1	B
3	1	1	−1	A	13	0.1	−1	0	A
3	1	1	1	C	13	1	1	−1	C
4	1	0	0.3	B	14	−1	1	0.2	A
4	−1	−1	1	C	14	1	1	1	C
4	−0.1	−1	−1	A	14	0.1	−1	1	B
4	−1	1	1	A	14	1	−1	−1	C
4	1	1	−1	C	14	−1	0	−1	B
5	−1	1	−1	C	15	−1	1	1	B
5	1	−1	1	C	15	1	1	−1	A
5	−0.1	1	1	A	15	1	−1	−1	B
5	−1	−1	0.2	B	15	−1	−1	1	A
5	1	−1	−1	A	15	−1	−1	−1	C
6	1	−1	−1	C	16	1	−1	1	B
6	1	−1	1	A	16	−1	1	−0.1	B
6	−1	1	−1	A	16	1	0.1	−1	C
6	0	1	1	C	16	−1	−1	−1	A
6	−1	−1	−1	B	16	1	1	1	A
7	−1	−1	1	B	17	1	0.1	1	C
7	−1	−1	−1	C	17	1	1	−1	B
7	1	−1	1	A	17	−1	1	−1	C
7	1	1	−1	B	17	−1	−1	1	B
7	−1	1	0.1	A	17	1	−1	−1	A
8	−1	−0.1	1	A	18	1	−1	1	B
8	−0.2	1	−1	C	18	−1	−1	−1	A
8	1	1	1	B	18	1	1	1	A
8	1	−1	1	C	18	−1	0	1	C
8	1	−1	−1	A	18	0	1	−1	B
9	−1	1	1	C	19	−1	−1	−1	B
9	1	−1	1	A	19	−1	1	−1	A
9	−1	−1	−1	C	19	1	1	0	B
9	1	1	−1	B	19	1	−1	1	A
9	−1	−1	1	B	19	−1	−1	1	C
10	1	−1	−1	C	20	−1	−1	−1	B
10	−1	1	−1	B	20	−1	1	−1	A
10	0	−1	1	B	20	−1	−0.1	1	C
10	−1	1	1	C	20	1	1	1	B
10	1	1	1	A	20	1	−1	−1	C

Using an *η*-value of 1, the relative efficiency of the original design in Table 5 to the *D*-optimal design is 91.5%. The relative efficiency of the design that was created by using the Trinca and Gilmour (2001) algorithm is 89.9%. The large improvements in efficiency are remarkable given the fact that the building-blocks in the two sequential approaches were obtained by using the *D*-optimality criterion as well. One could say that, compared with the *D*-optimal design, using the designs in Tables 5 and 6 results in a loss of nine and 10 observations respectively.

In addition to the factors that are related to the plasma treatment, the effect of several additives in the polypropylene was studied as well. This was because polypropylene is often compounded with additives such as stabilizers against ultraviolet (UV) light, lubricants, talc, mica and/or colour pigments to tailor the plastic to a specific end use. The engineers from the four companies strongly believed that some of these additives had an effect on the adhesive properties. This was because additives like UV stabilizer affect the surface of the polypropylene, which is exactly where the adhesion should take place. After a long debate, seven additives were included in the experiment: ethylene propylene diene monomer (EPDM) rubber, ethylene copolymer content of the rubber, talc, mica, lubricant, UV stabilizer and ethylene vinyl acetate. The ranges that were utilized for each of those factors are shown in Table 3, along with the ranges and levels of the four plasma treatment factors.

One multicomponent constraint had to be taken into account when designing the experiment: the additives talc and mica were not allowed to have strictly positive levels simultaneously. The reason for this is that talc and mica have similar effects on the properties of the polypropylene and are almost never used together when producing plastics.

## 7. Discussion

We have described a flexible algorithm for finding *D*-optimal split-plot designs. The algorithm runs in polynomial time with respect to the number of observations and number of factors. This is in contrast with algorithms that are based on a candidate set approach which run in exponential time in the number of factors.

We have also demonstrated the power of this tool through finding a previously unpublished optimal design. There are two ways in which the tool proposed can save practitioners money. First, the candidate-set-free algorithm provides an opportunity to reduce the sample size compared with the use of fractional factorial designs. Second, it allows the computation of more efficient solutions for challenging practical problems than can be found by using available methods, as in the polypropylene example.

There are a couple of opportunities for extending this work. An extension of the methodology to *I*-optimal designs and to multistratum designs is one possibility. Another straightforward extension would be to support Bayesian *D*-optimal designs as described in DuMouchel and Jones (1994) to reduce the dependence of these designs on the specification of an *a priori* model. Finally the need to discretize the range of each co-ordinate could be avoided by using an efficient one-dimensional non-linear optimization technique (see, for example, Brent (1973)). This modification would yield a continuous optimization while requiring only a minor change in the algorithm.

## Appendix A: The candidate-set-free algorithm

### A.1. Pseudocode

For clarity of explanation and simplicity of notation, we have assumed that all whole-plot sizes in the design are equal to *k* and that all factors are continuous. Also, we assumed that the values of *m*_*w*_ whole-plot factors have been arranged in the first *m*_*w*_ columns of the design matrix and the *m*_*s*_ subplot factors have been arranged in the columns from *m*_*w*_+1 to *m*_*w*_+*m*_*s*_.

The current best -criterion value found by the algorithm is denoted by _opt_. The current -criterion value during a try is denoted by . We assume that an appropriate discretization of the range for each continuous factor has been generated. The number of values for the discretized factor *i* is denoted by *L*_*i*_ and the set of values by *F*_*i*_={*f*_*i*1_,…,*f*_*iL*_*i*__}. Finally, we denote the number of tries by *t* and the number of the current try by *t*_c_.

*Step 1*: set _opt_=0 and *t*_c_=1.

*Step 2*: generate the starting design.

(a) Randomly generate values for the *m*_*w*_ whole-plot factors.

- Set *i*=1.
- Set *j*=1.
- Randomly generate a value for whole-plot factor *j* in whole plot *i*.
- Assign that value to rows *k*(*i*−1)+1 to *ki* of column *j* of the factor settings matrix.
- If *j*<*m*_*w*_, then set *j*=*j*+1 and go back to step 2, part (a) (iii).
- If *i*<*b*, then set *i*=*i*+1 and go back to step 2, part (a) (ii).

(b) Randomly generate levels for the *m*_*s*_ subplot factors.

- Set *i*=1.
- Set *j*=1.
- Randomly generate a value for subplot factor *j* in run *i*.
- Assign that value to cell (*i*,*m*_*w*_+*j*) of the design matrix.
- If *j*<*m*_*s*_, then set *j*=*j*+1 and go back to step 2, part (b) (iii).
- If *i*<*n*, then set *i*=*i*+1 and go back to step 2, part (b) (ii).

*Step 3*: compute the *D*-criterion value  of the starting design.

*Step 4*: improve the current design.

(a) Set *κ*=0.

(b) Set *i*=1.

(c) Improve whole-plot factor levels in whole plot *i*.

- Set *j*=1.
- Set *δ*=0.
- ∀*f*_*jl*_ ∈ *F*_*j*_:
  - replace the value of whole-plot factor *j* with *f*_*jl*_ in rows *k*(*i*−1)+1 to *ki* of the factor settings matrix;
  - compute the *D*-criterion value _*l*_ of the modified design;
  - if _*l*_>, then set *κ*=1, *δ*=1, =_*l*_ and *l*_max_=*l*.
- If *δ*=1, then replace the value of whole-plot factor *j* with the *l*_max_th element of *F*_*j*_ in rows *k*(*i*−1)+1 to *ki* of the factor settings matrix.
- If *j*<*m*_*w*_, then set *j*=*j*+1 and go back to step 4, part (c) (ii).

(d) Improve subplot factor levels for all *k* runs in whole plot *i*.

- Set *r*=1.
- Set *j*=1.
- Set *δ*=0.
- ∀*f*_*m*_*w*_+*j*,*l*_ ∈ *F*_*m*_*w*_+*j*_:
  - replace the value of subplot factor *j* with *f*_*m*_*w*_+*j*,*l*_ in cell (*k*(*i*−1)+*r*,*m*_*w*_+*j*) of the factor settings matrix;
  - compute the *D*-criterion value _*l*_ of the modified design;
  - if _*l*_>, then set *κ*=1, *δ*=1, =_*l*_ and *l*_max_=*l*.
- If *δ*=1, then replace the value of subplot factor *j* with *f*_*m*_*w*_+*j*,*l*_max__ in cell (*k*(*i*−1)+*r*,*m*_*w*_+*j*) of the factor settings matrix.
- If *j*<*m*_*s*_, then set *j*=*j*+1 and go back to step 4, part (d) (iii).
- If *r*<*k*, then set *r*=*r*+1 and go back to step 4, part (d) (ii).

*Step 5*: if *i*<*b*, then set *i*=*i*+1 and go back to step 4, part (c).

*Step 6*: if *κ*=1, then go back to step 4, part (a).

*Step 7*: if >_opt_, then set _opt_= and store the current design.

*Step 8*: if *t*_c_<*t*, then *t*_c_=*t*_c_+1 and go to step 2; otherwise stop.

### A.2. Categorical factors, mixture components and linear inequality constraints

For clarity of the pseudocode, we assumed that the factors were continuous. However, the candidate-set-free algorithm can handle categorical factors in much the same way as it copes with continuous factors. Mixture components can be handled also, but this is more involved as changing the proportion of one mixture component cannot be done independently. The way that we dealt with this complication makes use of the Cox effect direction and is described in Piepel *et al.* (2005). Linear inequality constraints on the factors can be handled by using a flexible discretization over the feasible range of each factor, given the values of the other factors.
